# Stochastic Thermodynamics of a Finite Quantum System Coupled to Two Heat Baths

**DOI:** 10.3390/e25030504

**Published:** 2023-03-15

**Authors:** Heinz-Jürgen Schmidt, Jochen Gemmer

**Affiliations:** Fachbereich Physik, Universität Osnabrück, D-49069 Osnabrück, Germany

**Keywords:** heat conduction, fluctuation theorems, Clausius relation

## Abstract

We consider a situation where an *N*-level system (NLS) is coupled successively to two heat baths with different temperatures without being necessarily thermalized and approaches a steady state. For this situation we apply a general Jarzynski-type equation and conclude that heat and entropy is flowing from the hot bath to the cold one. The Clausius relation between increase of entropy and transfer of heat divided by a suitable temperature assumes the form of two inequalities. Our approach is illustrated by an analytical example. For the linear regime, i.e., for small temperature differences between the two heat baths, we derive an expression for the heat conduction coefficient.

## 1. Introduction

The study of the non-equilibrium thermodynamics of heat conduction has a long history. As an example, consider the sequence of papers of Lebowitz and Spohn [1,2,3] dealing with both classical and quantum aspects of this problem. A considerable number of papers concerned with thermal conduction for quantum systems consider a kind of chain of small quantum systems connected to the two heat baths at their ends and focus on the questions of whether a local thermal equilibrium is reached in the small quantum systems, whether a constant temperature gradient exists, and whether the heat transfer rate is proportional to this gradient (“Fourier’s law”); see, e.g., [4,5,6,7,8,9,10,11].

The present paper is *not* concerned with the validity of Fourier’s law for special systems, but rather assumes a general *N*-level system (NLS) with alternating contact with two heat baths and studies the heat and entropy transfer in the steady state assumed after some time, without further analyzing the internal structure of the NLS. For this purpose, we use new methods that have been developed during the last decades, in particular, the approach via fluctuation theorems; see, e.g., [3]. The famous Jarzynski equation represents one of the rare exact results in nonequilibrium statistical mechanics. It is originally a statement of the expectation value of the exponential of the work $\langle e^{-\beta \;w}\rangle$ performed on a system initially in thermal equilibrium with inverse temperature β, but may be far from equilibrium after the work process. This equation was first formulated for classical systems [12,13,14] and subsequently proved for quantum systems [15,16,17,18]. Extensions for systems initially in local thermal equilibrium [17], micro-canonical ensembles [19], and grand-canonical ensembles [20,21,22,23,24,25,26] have been published.

The most common interpretation of the quantum Jarzynski equation is to consider sequential measurements; see [15]. However, there are recent alternative approaches to obtain fluctuation relations. We note the derivation of fluctuation theorems for heat exchange in a quantum correlated bipartite thermal systems using the framework of dynamic Bayesian networks [27], the Margenau–Hill scheme [28], and the end-point-measurement scheme [29].

Nevertheless, in the present work we adopt the sequential measurements approach. The general framework for such an approach was outlined in [30,31]. It is neither quantum mechanical nor classical per se and will be referred to as “stochastic thermodynamics” in the present work. We recently applied this method to the problem of the interaction of an NLS with a single heat bath [32]. Here, we extend this work to the interaction with two heat baths of possibly different temperatures. Again, it is possible to describe the influence of the heat baths by an N×N stochastic matrix *T* that gives the conditional probability of transitions between the *N* levels of the NLS. Moreover, it is sensible to identify the steady state regime with fixed points of *T*.

**Figure 1:**
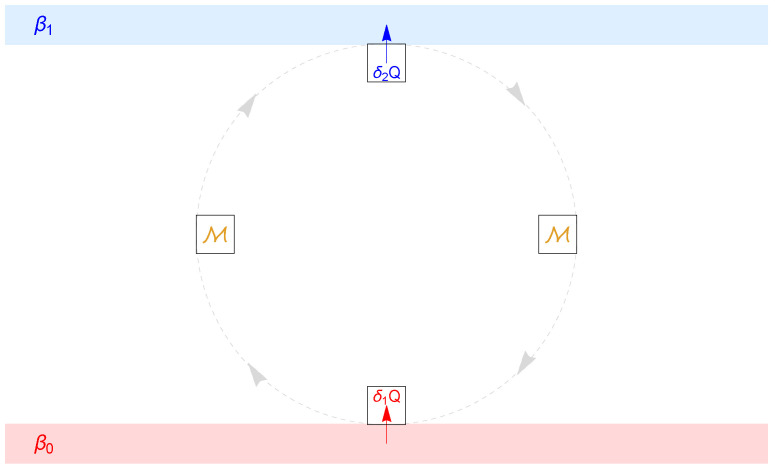
Schematic sketch of one cycle of the alternate interaction of the NLS (black squares) with two heat baths of inverse temperature $\beta_{0}<\beta_{1}$, resp., interspersed with energy measurements (yellow M). At steady state, the heat $\delta_{1}Q$ absorbed by the NLS during contact with the first heat bath equals in amount the heat $\delta_{2}Q$ emitted during contact with the second heat bath.

However, it is not readily possible to infer the heat flow between the NLS and heat baths from the change in energy of the NLS, analogously for the entropy flow. At steady state, the corresponding changes of energy and entropy would vanish, although the flow may be non-zero. To cope with this problem we consider a step-wise interaction with the heat baths; see Figure 1. We start with a preparatory energy measurement and a contact only with the first heat bath, followed by a second energy measurement, together described by a stochastic matrix $T^{\left(0\right)}$. This is followed by a separate contact with the second heat bath and another measurement, together described by $T^{\left(1\right)}$. The total interaction plus measurements is hence given by the product matrix $T=T^{\left(1\right)}\;T^{\left(0\right)}$. Moreover, we will assume, similarly as in [32], that the single stochastic matrices $T^{\left(i\right)}$ have invariant probability distributions of Gibbs type, thereby introducing the two inverse temperatures $\beta_{i},\;i=0,1$ of the heat baths. This enables us to investigate the dependance between direction of heat and entropy flow and the temperature difference, at least for the steady state. The main message is that, although it would be very difficult to calculate the stochastic matrices $T^{\left(0\right)}$ and $T^{\left(1\right)}$ for “real” systems, the validity of the second law-like statement about the correct direction of heat and entropy flow only depends on some general properties of these matrices and not of their particular entries.

Our approach thus fits into the framework of collision models, also known as repeated interaction schemes. They are based on the idea of discretizing reduced dynamics in time and simplifying the environment as a collection of identical and independent entities. For a recent account on collision models, see [33]. A representation of sequential heat exchange similar to ours was given in [34] where, however, fluctuations theorems of Crooks’ type [35] were considered.

The paper is organized as follows. In Section 2, we present the pertinent definitions and assumptions that characterize the present approach to the heat conduction problem. The main result is obtained in Section 3, where we formulate the Jarzynski-type equation suited for the present problem and apply Jensen’s inequality to derive two Clausius inequalities. These inequalities immediately imply that, in the steady state and in the statistical mean, heat and entropy always flow from the hotter to the colder bath. Section 4 is devoted to two analytical examples where the heat and entropy flows noted above can be explicitly calculated by computer-algebraic means. In the first example, we consider stochastic matrices $T^{\left(0\right)}$ and $T^{\left(1\right)}$ describing partial thermalization. In the second example, a three-level system is alternately coupled to two harmonic oscillators with different temperatures, and the interaction between the system and the harmonic oscillators is described analogously to the Jaynes–Cummings model. Moreover, in this example, the steady state $p^{\left(s\right)}$ is characterized by two parameters $\beta^{\left(s\right)}$ and $\gamma^{\left(s\right)}$, $\beta^{\left(s\right)}$ being some sort of inverse temperature and $\gamma^{\left(s\right)}$ characterizing the deviation of the steady state from a Gibbs state, and the dependence of $\beta^{\left(s\right)}$ and $\gamma^{\left(s\right)}$ on the inverse temperatures of the two heat baths can be explicitly determined. In the linear regime $\beta_{0}\approx \beta_{1}$, considered in Section 5, the steady state $p^{\left(s\right)}$ will only infinitesimally deviate from the Gibbs state of inverse temperature $\beta_{0}$. This deviation can be calculated by means of first order perturbation theory and used to determine the common slope *a* of the functions of entropy transfer and (reduced) heat transfer at $\beta_{1}=\beta_{0}$. The result, of course, depends of the eigenvalues and eigenvectors of $T^{\left(0\right)}$, which can be obtained for the analytical example of Section 4.2 but probably not, or only approximately, for “real” systems. We close with a summary and outlook in Section 6.

## 2. Basic Definitions

We consider an *N*-level system (NLS) described by a finite index set N, energies $E_{n}$, and degeneracies $d_{n}$ for n∈N. The Hamiltonian for the NLS without interaction with the heat baths will hence be of the form

(1) HH∼=∑nEnPP∼n,

with a complete family of mutually orthogonal projections PP∼n,n∈N, and dn=TrPP∼n.

The Gibbs states for the NLS with inverse temperature β>0 are characterized by probabilities

(2) $$p_{n}^{\left(\beta \right)}=\frac{d_{n}}{Z^{\left(\beta \right)}}\;\exp\left(-\beta \;E_{n}\right)\;,$$

where the partition function $Z^{\left(\beta \right)}$ is given by

(3) $$Z^{\left(\beta \right)}=\sum_{n}d_{n}\;\exp\left(-\beta \;E_{n}\right)\;.$$

We assume a rather general initial state ρρ∼(i) of the NLS of the form

(4) ρρ∼(i)=∑npn(i)PP∼ndn,

which is not necessarily a Gibbs state. After the interaction with the first heat bath, we perform a Lüders measurement of the energy that, without selection according to the outcome, is assumed to yield the intermediate mixed state

(5) ρρ∼′=∑nqn(i)PP∼ndn,

such that the transition between the probability distributions $p^{\left(i\right)}\mapsto q^{\left(i\right)}$ is given by a left stochastic matrix $T^{\left(0\right)}$ characterizing the first heat bath according to

(6) $$q_{n}^{\left(i\right)}=\sum_{m}T_{nm}^{\left(0\right)}\;p_{m}^{\left(i\right)},\;\mathrm{for}\mathrm{all}n\in N\;.$$

Thereafter, an interaction with a second heat bath is assumed that, after a second Lüders measurement of the energy, results in the final state

(7) ρρ∼(f)=∑npn(f)PP∼ndn,

such that, analogously to the first step,

(8) $$p_{n}^{\left(f\right)}=\sum_{m}T_{nm}^{\left(1\right)}\;q_{m}^{\left(i\right)},\;\mathrm{for}\mathrm{all}n\in N\;,$$

where $T^{\left(1\right)}$ is another (left) stochastic matrix characterizing the second heat bath.

If we model the heat baths by quantum systems and their successive interaction with the NLS by some unitary time evolution, the forms (5) and (7) of the intermediate state and the final state of the NLS does not follow automatically but represents a crucial assumption of our approach. Only if the NLS is non-degenerate, i.e., all $d_{n}=1$, then (5) and (7) will follow from general principles. In other cases, these assumptions may fail or be only approximately satisfied.

The stochastic matrices $T^{\left(0\right)}$ and $T^{\left(1\right)}$ generally possess invariant probability distributions or *fixed points*, i.e., eigenvectors corresponding to the eigenvalue 1 with non-negative entries. At the moment, we need not assume that these probability distributions are unique, but we will rather postulate that there exist invariant probability distributions of Gibbs type, i.e.,

(9) $$\sum_{m}T_{nm}^{\left(i\right)}\;p_{m}^{\left(\beta_{i}\right)}=p_{n}^{\left(\beta_{i}\right)},\;\mathrm{for}\mathrm{all}n\in N\mathrm{and}i=0,1\;.$$

It will be plausible to identify the inverse temperatures $\beta_{0}$ and $\beta_{1}$ with the respective inverse temperatures of the heat baths. According to the zeroth law of thermodynamics the interaction between a heat bath and an NLS of the same temperature should not change the occupation probabilities of the energy levels.

The total transition $p^{\left(i\right)}\mapsto q^{\left(i\right)}\mapsto p^{\left(f\right)}$ will be achieved by the product matrix

(10) $$T=T^{\left(1\right)}\;T^{\left(0\right)}\;,$$

which is again a stochastic matrix. In the case of $\beta_{0}=\beta_{1}$, the Gibbs state probabilities $p_{n}^{\left(\beta_{0}\right)}$ would form an invariant probability distribution of *T*, but in the general case of $\beta_{0}\neq \beta_{1}$ the fixed point of *T* (not necessarily unique) will be different from $p^{\left(\beta_{i}\right)},\;i=0,1$. Starting with an arbitrary initial distribution $p^{\left(i\right)}$ and iterating the above-described protocol of successive interactions with the heat baths and energy measurements, one would obtain a sequence $T^{\nu }p^{\left(i\right)}$ of probability distributions that converge towards a fixed point of *T*:

(11) $$p^{\left(s\right)}=\lim_{\nu \to \infty }T^{\nu }p^{\left(i\right)}\;.$$

If the fixed point is not unique, $p^{\left(s\right)}$ will depend on the initial distribution $p^{\left(i\right)}$. Physically, $p^{\left(s\right)}$ represents a *steady state* that will be asymptotically assumed. The NLS transports heat and entropy between the two heat baths, which are approximately unchanged (otherwise the stochastic matrix *T* could not be kept constant); see Figure 1. This is similar as for the Carnot cycle of classical thermodynamics. The difference, however, is that we do not consider additional extraction of work and allow for irreversible processes, whereas the Carnot process is reversible.

The steady state distribution $p^{\left(s\right)}$ can be used to calculate the steady flow of heat and entropy between the two heat baths due to the process described above. A natural question is whether this flow is always directed from the hotter bath to the colder one, as one would expect in accordance with the second law of thermodynamics. This question will be addressed in Section 3.

## 3. Direction of Heat and Entropy Flow

### 3.1. General Fluctuation Theorem

In order to make this paper as self-contained as possible, we will recapitulate the scenario of stochastic thermodynamics and the derivation of a general fluctuation theorem considered in [30,31,32] in a somewhat simplified form.

We consider two subsequent measurements at a physical system with finite outcome sets, I for the first and J for the second measurement. The total event space will hence be I×J, and the statistics of the subsequent measurements will be given by a probability distribution

(12) P:I×J→[0,1],

hence satisfying

(13) $$\sum_{ij}P\left(i,j\right)=1\;.$$

We define the “initial probability”

(14) $$p_{i}:=\sum_{j}P\left(i,j\right)\;,$$

and the (left) stochastic “transition matrix” T with entries

(15) $$T_{ji}:=\frac{P(i,j)}{p_{i}}\;,$$

assuming that $p_{i}>0$ for all i∈I. It follows that the “final probabilities” are given by

(16) $$q_{j}:=\sum_{i}P\left(i,j\right)=\sum_{i}T_{ji}\;p_{i}\;.$$

Further consider two arbitrary probability distributions $\tilde{p}:J\to \left[0,1\right]$ and $p^{\left(0\right)}:I\to \left[0,1\right]$ and define $q^{\left(0\right)}:J\to \left[0,1\right]$ by

(17) $$q_{j}^{\left(0\right)}:=\sum_{i}T_{ji}\;p_{i}^{\left(0\right)}\;.$$

Then the general fluctuation theorem can be formulated as

(18) $$\langle \frac{p_{i}^{\left(0\right)}\;{\tilde{p}}_{j}}{q_{j}^{\left(0\right)}\;p_{i}}\rangle =1\;.$$

Here, the terms in the bracket represent four random variables indicated by their values, and the bracket indicates the expectation value to be evaluated by means of the probability distribution (12). The proof of (18) is surprisingly simple:

(19) $$\begin{array}{ccc} \langle \frac{p_{i}^{\left(0\right)}\;{\tilde{p}}_{j}}{q_{j}^{\left(0\right)}\;p_{i}}\rangle & = & \sum_{ij}P\left(i,j\right)\;\frac{p_{i}^{\left(0\right)}\;{\tilde{p}}_{j}}{q_{j}^{\left(0\right)}\;p_{i}} \end{array}$$

(20) $$\begin{array}{ccc} & \overset{\left(15\right)}{=} & \sum_{ij}T_{ji}\;\frac{p_{i}^{\left(0\right)}\;{\tilde{p}}_{j}}{q_{j}^{\left(0\right)}} \end{array}$$

(21) $$\begin{array}{ccc} & \overset{\left(17\right)}{=} & \sum_{j}\frac{q_{j}^{\left(0\right)}\;{\tilde{p}}_{j}}{q_{j}^{\left(0\right)}}=\sum_{j}{\tilde{p}}_{j}=1\;. \end{array}$$

The general fluctuation theorem (18) is more of a template that provides sensible statements only when the arbitrary probability distributions $\tilde{p}$ and $p^{\left(0\right)}$ have been suitably chosen. We will, in the next subsection, show how the famous Jarzynsiki equation results as a special case of (18) and then consider the choices that yield useful results for our problem.

### 3.2. Jarzynski Equation

We consider two subsequent energy measurements at a *d*-dimensional quantum system corresponding to two Hamiltonians with spectral decompositions

(22) HH∼(t0)=∑iEiPP∼i,

(23) HH∼(t1)=∑jE˜jQQ∼j,

not necessarily commuting and with degeneracies of eigenspaces

(24) di=TrPP∼i,satisfying∑idi=d,

(25) Dj=TrQQ∼j,satisfying∑jDj=d.

Let

(26) $$\begin{array}{ccc} Z & = & \sum_{i}d_{i}\exp\left(-\beta E_{i}\right)=:e^{-\beta F}\;, \end{array}$$

(27) $$\begin{array}{ccc} \tilde{Z} & = & \sum_{j}D_{j}\exp\left(-\beta {\tilde{E}}_{j}\right)=:e^{-\beta \tilde{F}} \end{array}$$

be the corresponding partition functions. Between the two measurements, a unitary time evolution *U* takes place (not necessarily corresponding to H(t)), and the initial probability distribution is assumed to be a Gibbs state with inverse temperature β. Hence the total probability distribution *P* is given by

(28) P(i,j)=TrQQ∼jUU∼PP∼idiUU∼*︸Tjidiexp−βEiZ︸pi.

Then we make the choices

(29) $$p_{i}^{\left(0\right)}:=d_{i}/d\;,$$

which, after a short calculation, leads to

(30) $$q_{j}^{\left(0\right)}=D_{j}/d\;,$$

and

(31) $${\tilde{p}}_{j}:=\frac{D_{j}\;\exp\left(-\beta {\tilde{E}}_{j}\right)}{\tilde{Z}}\;.$$

With these choices, (18) assumes the form

(32) $$\begin{array}{ccc} 1 & = & \langle \frac{d_{i}}{D_{j}}\frac{D_{j}\exp\left(-\beta {\tilde{E}}_{j}\right)}{\tilde{Z}}\frac{Z}{d_{i}\exp\left(-\beta E_{i}\right)}\rangle \end{array}$$

(33) $$\begin{array}{ccc} & \overset{\left(26),(27\right)}{=} & \langle e^{-\beta \left({\tilde{E}}_{j}-E_{i}\right)}\rangle \;e^{-\beta \left(F-\tilde{F}\right)}\;. \end{array}$$

From this, the usual form of the Jarzynski equation

(34) $$\langle e^{-\beta \;w}\rangle =e^{-\beta \;\Delta F}$$

immediately follows if we define the random variable w:I×J→R (“work”) by

(35) $$w\left(i,j\right):={\tilde{E}}_{j}-E_{i}\;,$$

and denote the difference of free energies by $\Delta F:=\tilde{F}-F$.

### 3.3. Fluctuation Theorem and Direction of Heat and Entropy Flow

We retain the basic definitions of Section 2 and make the following choices in order to apply the general fluctuation theorem in Section 3.1. First, we identify the outcome sets of the two energy measurements with the level set of the NLS: I=J=N.

Then we will apply (18) in two ways: The first one is given by

(36) $$T=T^{\left(0\right)},\;p_{n}=p_{n}^{\left(s\right)},\;{\tilde{p}}_{m}=q_{m}^{\left(s\right)}=\sum_{n}T_{mn}^{\left(0\right)}p_{n}^{\left(s\right)}\mathrm{and}p_{n}^{\left(0\right)}=p_{n}^{\left(\beta_{0}\right)}\;.$$

It follows that

(37) $$q_{m}^{\left(0\right)}=\sum_{n}T_{mn}\;p_{n}^{\left(0\right)}=\sum_{n}T_{mn}^{\left(0\right)}\;p_{n}^{\left(\beta_{0}\right)}=p_{m}^{\left(\beta_{0}\right)}$$

according to (9), and the fluctuation theorem (18) assumes the form:

(38) $$\langle \frac{p_{n}^{\left(\beta_{0}\right)}\;q_{m}^{\left(s\right)}}{p_{m}^{\left(\beta_{0}\right)}\;p_{n}^{\left(s\right)}}\rangle =1\;.$$

Using Jensen’s inequality (JI) and the fact that x↦−logx is a convex function, we conclude, analogously to [32] ((63)–(66)),

(39) $$\begin{array}{ccc} 0 & = & -\log1\overset{\left(38\right)}{=}-\log\langle \frac{p_{n}^{\left(\beta_{0}\right)}\;q_{m}^{\left(s\right)}}{p_{m}^{\left(\beta_{0}\right)}\;p_{n}^{\left(s\right)}}\rangle \end{array}$$

(40) $$\begin{array}{ccc} & \overset{\left(JI\right)}{\leq } & \langle -\log\frac{p_{n}^{\left(\beta_{0}\right)}}{d_{n}}+\log\frac{p_{m}^{\left(\beta_{0}\right)}}{d_{m}}-\log\frac{q_{m}^{\left(s\right)}}{d_{m}}+\log\frac{p_{n}^{\left(s\right)}}{d_{n}}\rangle \end{array}$$

(41) $$\begin{array}{ccc} & = & \langle \beta_{0}E_{n}+\log Z^{\left(\beta_{0}\right)}-\beta_{0}E_{m}-\log Z^{\left(\beta_{0}\right)}\rangle -\sum_{m}q_{m}^{\left(s\right)}\;\log\frac{q_{m}^{\left(s\right)}}{d_{m}}+\sum_{n}p_{n}^{\left(s\right)}\;\log\frac{p_{n}^{\left(s\right)}}{d_{n}} \end{array}$$

(42) $$\begin{array}{ccc} & = & \langle \Delta S\rangle -\beta_{0}\langle \Delta Q\rangle \;. \end{array}$$

Here we have used the obvious definitions of the random variables “heat transfer”

(43) $$\Delta Q\left(m,n\right):=E_{m}-E_{n}\;,$$

and “entropy transfer”

(44) $$\Delta S\left(m,n\right):=\log\frac{p_{n}}{d_{n}}-\log\frac{q_{m}}{d_{m}}\;.$$

Hence we have shown the following *Clausius inequality*:

(45) $$\langle \Delta S\rangle \geq \beta_{0}\langle \Delta Q\rangle \;.$$

Note that the choice $p_{n}=p_{n}^{\left(s\right)}$ is not necessary to derive (45); similarly as in [32], this Clausius inequality would hold for arbitrary initial probability distributions. However, the choice of a steady state distribution will be crucial to derive the second Clausius inequality; see (54) below.

The second way of applying (18) will be given by the choice

(46) $$\begin{array}{ccc} T & = & T^{\left(1\right)},\;p_{n}=\sum_{m}T_{nm}^{\left(0\right)}\;p_{m}^{\left(s\right)}, \end{array}$$

(47) $$\begin{array}{ccc} {\tilde{p}}_{m} & = & q_{m}=\sum_{n}T_{mn}p_{n}=\sum_{n\ell }T_{mn}^{\left(1\right)}T_{n\ell }^{\left(0\right)}p_{\ell }^{\left(s\right)}=\sum_{\ell }T_{m\ell }p_{\ell }^{\left(s\right)}=p_{m}^{\left(s\right)}\;, \end{array}$$

(48) $$\begin{array}{ccc} p_{n}^{\left(0\right)} & = & p_{n}^{\left(\beta_{1}\right)}\;. \end{array}$$

Again, $q_{m}^{\left(0\right)}=\sum_{n}T_{mn}^{\left(1\right)}p_{n}^{\left(\beta_{1}\right)}=p_{m}^{\left(\beta_{1}\right)}$ holds according to (9). Due to the fixed point property of $p^{\left(s\right)}$ with respect to $T=T^{\left(1\right)}\;T^{\left(0\right)}$, the choice (46)–(48) implies that the roles of $p_{n}$ and $q_{m}$ are interchanged. This means that, at steady state, the NLS absorbs the same amount of heat during contact with the first heat bath as it emits during the contact with the second one, analogously for the entropy. It follows that (45) again holds, but with the replacements

(49) $$\begin{array}{ccc} \langle \Delta S\rangle & \mapsto & -\langle \Delta S\rangle , \end{array}$$

(50) $$\begin{array}{ccc} \langle \Delta Q\rangle & \mapsto & -\langle \Delta Q\rangle ,\;\mathrm{and} \end{array}$$

(51) $$\begin{array}{ccc} \beta_{0} & \mapsto & \beta_{1}\;. \end{array}$$

This gives

(52) $$\begin{array}{ccc} -\langle \Delta S\rangle & \geq & -\beta_{1}\langle \Delta Q\rangle \;, \end{array}$$

(53) $$\begin{array}{ccc} \langle \Delta S\rangle & \leq & \beta_{1}\langle \Delta Q\rangle \;, \end{array}$$

and thus, together with (45), we obtain the *Clausius inequalities*

(54) $$\beta_{0}\langle \Delta Q\rangle \leq \langle \Delta S\rangle \leq \beta_{1}\langle \Delta Q\rangle \;.$$

These inequalities have the same form as the corresponding inequalities derived in [32] and, under different assumptions, in [36,37,38,39,40,41], but generally a different meaning. For example, in [32] the inverse temperature $\beta_{1}$ refers to the NLS before the interaction with the heat bath of inverse temperature $\beta_{0}$, but in (54) the inverse temperature $\beta_{1}$ refers to the second heat bath, whereas the NLS is in the steady state and, in general, has no temperature at all.

Immediate consequences of (54) are

(55) $$\left(\beta_{1}-\beta_{0}\right)\langle \Delta Q\rangle \geq 0\;,$$

and

(56) $$\begin{array}{ccc} \left(\beta_{1}-\beta_{0}\right)\langle \Delta S\rangle & = & \beta_{1}\;\langle \Delta S\rangle -\beta_{0}\;\langle \Delta S\rangle \end{array}$$

(57) $$\begin{array}{ccc} & \overset{\left(54\right)}{\geq } & \beta_{1}\left(\beta_{0}\langle \Delta Q\rangle \right)-\beta_{0}\left(\beta_{1}\langle \Delta Q\rangle \right) \end{array}$$

(58) $$\begin{array}{ccc} & = & 0\;. \end{array}$$

These equations imply that, in the steady state regime and in the statistical mean, heat and entropy always flow from the hotter bath to the colder one.

## 4. Analytical Examples

### 4.1. Analytical Example I: Partial Thermalization

We will consider an example where all considered quantities can be analytically calculated for arbitrary *N*. The stochastic matrices $T^{\left(0\right)}$ and $T^{\left(1\right)}$ are not obtained by studying the interaction with a heat bath but are chosen phenomenologically to represent a mixture of perfect thermalized and an unchanged distribution. This choice obviously satisfies postulate (9). For simplicity, we consider only the case of a mixture with equal proportions. The *N* energies of the NLS are chosen as $E_{n}=n$ for n=1,…,N, without degeneracy.

The stochastic matrix P(β) of perfect thermalization is given by

(59) $$P{\left(\beta \right)}_{nm}=\frac{1}{Z(\beta )}\exp\left(-\beta \;n\right)\;,$$

and hence

(60) $$T_{nm}^{\left(i\right)}=\frac{1}{2}\left(\delta_{nm}+P{\left(\beta_{i}\right)}_{nm}\right)\;,$$

for i=0,1. After some calculations, we obtain for the steady state distribution $p^{\left(s\right)}$, the fixed point of $T=T^{\left(1\right)}\;T^{\left(0\right)}$:

(61) $$p_{k}^{\left(s\right)}=\frac{1}{3}\left(\frac{\left(1-e^{\beta_{0}}\right)e^{\beta_{0}\left(N-k\right)}}{1-e^{\beta_{0}N}}+\frac{2\left(1-e^{\beta_{1}}\right)e^{\beta_{1}\left(N-k\right)}}{1-e^{\beta_{1}N}}\right)\;,$$

for k=1,…,N. This is a certain temperature-dependent mixture between the Gibbs states of inverse temperatures $\beta_{0}$ and $\beta_{1}$ of the two heat baths, which appears plausible.

**Figure 2:**
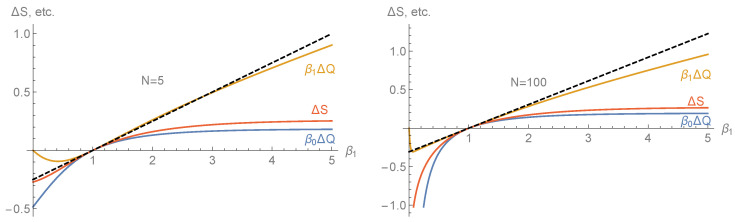
Plot of the transferred entropy $\langle \Delta S\rangle$ per cycle (red curves) and reduced heat transfer $\beta_{0}\;\langle \Delta Q\rangle$ (blue curves) and $\beta_{1}\;\langle \Delta Q\rangle$ (dark yellow curves) as a function of the inverse temperature $\beta_{1}$ of the second heat bath and fixed inverse temperature $\beta_{0}=1$ of the first one. The calculations have been made for the first analytical example of Section 4.1 and N=5 levels (left panel) as well as N=100 levels (right panel) and for the steady state regime. The common tangent at $\beta_{1}=\beta_{0}=1$ is indicated by dashed black lines with slope a≈0.249977(N=5), resp., a≈0.306891(N=100), according to the calculations in Section 5. Note that the Clausius inequalities (54) are satisfied and that the signs of $\langle \Delta S\rangle$ and $\langle \Delta Q\rangle$ coincide with the sign of $\beta_{1}-\beta_{0}$ in accordance with the second law of thermodynamics.

Using the steady state distribution and computer-algebraic means, we can calculate the various transferred quantities as $\Delta S,\beta_{i}\;\Delta Q$ for any given *N*, although the result is generally too complex to be shown here. These quantities are plotted in Figure 2 for the two cases of N=5 and N=100 energy levels, thereby confirming the Clausius inequalities (54) and the direction of the heat and entropy flow from the hotter to the colder bath.

### 4.2. Analytical Example II: Modified Jaynes–Cummings Model

As an example where the time average of the transition matrix T(β) corresponding to a heat bath of inverse temperature β can be exactly calculated, we have considered in [32] a single spin with spin quantum number s=1 coupled to a harmonic oscillator that serves as a heat bath. Hence we have a N=3-level system with N={1,0,−1}. The total Hamiltonian and further details are given in [32] and need not be repeated here. The Hamiltonian strongly resembles the Jaynes–Cummings model [42,43] originally describing the interaction of a 2-level system with a quantized radiation field but also considered for 3-level systems [44,45]. We reproduce the analytical result [32] for the entries of T(β):

(62) $$\begin{array}{ccc} T_{1,1}\left(\beta \right) & = & \frac{1}{32}\left(e^{\beta }-1\right)\left(\frac{12}{e^{\beta }-1}+8e^{\frac{\beta }{2}}\coth^{-1}\left(e^{\frac{\beta }{2}}\right)+3e^{-\beta }\Phi \left(e^{-\beta },2,\frac{3}{2}\right)-8\right), \end{array}$$

(63) $$\begin{array}{ccc} T_{1,0}\left(\beta \right) & = & \frac{1}{4}\left(1-2\sinh\left(\frac{\beta }{2}\right)\tanh^{-1}\left(e^{-\frac{\beta }{2}}\right)\right), \end{array}$$

(64) $$\begin{array}{ccc} T_{1,-1}\left(\beta \right) & = & \frac{3}{32}e^{-3\beta 0}\left(4e^{\beta }-\left(e^{\beta }-1\right)\Phi \left(e^{-\beta },2,\frac{3}{2}\right)\right), \end{array}$$

(65) $$\begin{array}{ccc} T_{0,1}\left(\beta \right) & = & \frac{1}{4}e^{\beta }\left(1-2\sinh\left(\frac{\beta }{2}\right)\tanh^{-1}\left(e^{-\frac{\beta }{2}}\right)\right), \end{array}$$

(66) $$\begin{array}{ccc} T_{0,0}\left(\beta \right) & = & \frac{1}{2}, \end{array}$$

(67) $$\begin{array}{ccc} T_{0,-1}\left(\beta \right) & = & \frac{1}{4}e^{-\frac{3\beta }{2}}\left(e^{\frac{\beta }{2}}+\left(e^{\beta }-1\right)\tanh^{-1}\left(e^{-\frac{\beta }{2}}\right)\right), \end{array}$$

(68) $$\begin{array}{ccc} T_{-1,1}\left(\beta \right) & = & \frac{3}{32}e^{-\beta }\left(4e^{\beta }-\left(e^{\beta }-1\right)\Phi \left(e^{-\beta },2,\frac{3}{2}\right)\right), \end{array}$$

(69) $$\begin{array}{ccc} T_{-1,0}\left(\beta \right) & = & \frac{1}{4}\left(2\sinh\left(\frac{\beta }{2}\right)\tanh^{-1}\left(e^{-\frac{\beta }{2}}\right)+1\right),\\ T_{-1,-1}\left(\beta \right) & = & \frac{1}{32}e^{-3\beta }\left(4e^{2\beta }\left(11\sinh\left(\beta_{0}\right)+5\cosh\left(\beta \right)-4\sinh\left(\frac{\beta }{2}\right)\coth^{-1}\left(e^{\frac{\beta }{2}}\right)-2\right)\right. \end{array}$$

(70) $$\begin{array}{ccc} & & \left.+3\left(e^{\beta }-1\right)\Phi \left(e^{-\beta },2,\frac{3}{2}\right)\right)\;. \end{array}$$

Here $\Phi \left(z,s,a\right):=\sum_{k\in N}\frac{z^{k}}{{\left(k+a\right)}^{s}}$ denotes Lerch’s transcendent; see [46] §25.14. It can be shown that T(β) is a left stochastic matrix and leaves the probability distribution

(71) $$p^{\left(\beta \right)}=\frac{1}{e^{-\beta }+1+e^{\beta }}\left(e^{-\beta },1,e^{\beta }\right)$$

invariant that corresponds to a Gibbs state with inverse temperature β.

**Figure 3:**
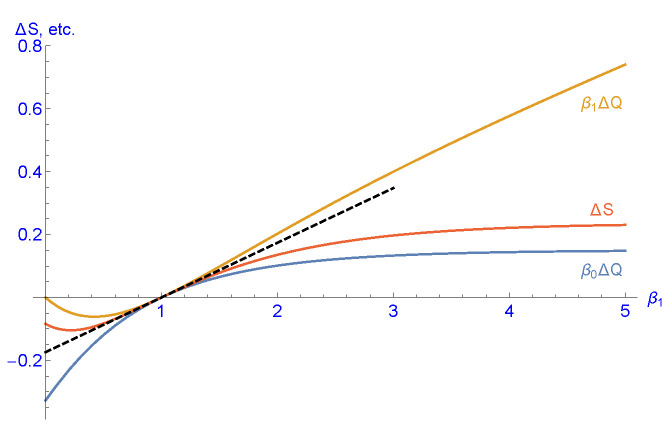
Plot of the transferred entropy $\langle \Delta S\rangle$ per cycle (red curve) and reduced heat transfer $\beta_{0}\;\langle \Delta Q\rangle$ (blue curve) and $\beta_{1}\;\langle \Delta Q\rangle$ (dark yellow curve) as a function of the inverse temperature $\beta_{1}$ of the second heat bath and fixed inverse temperature $\beta_{0}=1$ of the first one. The common tangent at $\beta_{1}=\beta_{0}=1$ is indicated by the dashed black line with slope a≈0.173895 that will be calculated in Section 5. The calculations have been made for the analytical example of Section 4.2 and steady state regime. Note that the Clausius inequalities (54) are satisfied and that the signs of $\langle \Delta S\rangle$ and $\langle \Delta Q\rangle$ coincide with the sign of $\beta_{1}-\beta_{0}$ in accordance with the second law of thermodynamics.

Using this analytical result, we set $T^{\left(0\right)}=T\left(\beta_{0}\right)$ and $T^{\left(1\right)}=T\left(\beta_{1}\right)$ for the model with two heat baths and can explicitly calculate the heat and entropy transfer $\langle \Delta S\rangle \left(\beta_{1}\right)$ and $\beta_{i}\;\langle \Delta Q\rangle \left(\beta_{1}\right)$, considered in Section 3, as functions of $\beta_{1}$. Although the result is too complex to be shown here, these transfer functions can be plotted for fixed values of $\beta_{0}$, say, $\beta_{0}=1$; see Figure 3. Thus one can graphically confirm the Clausius inequalities (54) and the correct sign of heat and entropy flow. The three functions have a common tangent at $\beta_{0}=\beta_{1}$, the slope of which will be calculated in Section 5.

In the limit β→0, we obtain the bi-stochastic matrix

(72) $$\lim_{\beta \to 0}T\left(\beta \right)=\frac{1}{8}\left(\begin{array}{ccc} 3 & 2 & 3\\ 2 & 4 & 2\\ 3 & 2 & 3 \end{array}\right)\;,$$

whereas for β→∞

(73) $$\lim_{\beta \to \infty }T\left(\beta \right)=\frac{1}{6}\left(\begin{array}{ccc} 3 & 0 & 0\\ 1 & 3 & 0\\ 2 & 3 & 6 \end{array}\right)\;.$$

Both limits facilitate the analytical calculation of corresponding asymptotic values of the pertinent functions. Of note, the latter one (73) is an example of a stochastic matrix where the algebraic and geometric degeneracy of the eigenvalues no longer coincide: the eigenvalue $t_{2}=1/2$ is doubly degenerate but possesses only a one-dimensional eigenspace $R\;{\left(0,1,-1\right)}^{⊤}$.

**Figure 4:**
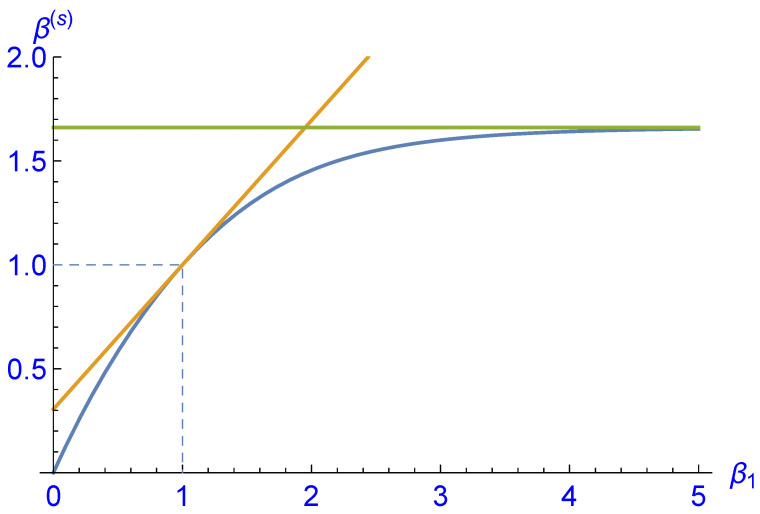
Plot of the first parameter $\beta^{\left(s\right)}$ characterizing the steady state distribution $p^{\left(s\right)}$ of the analytical example as a function of $\beta_{1}$ for fixed $\beta_{0}=1$ (blue curve). The slope $a_{1}=0.694846\ldots$ of the tangent at $\beta_{1}=\beta_{0}$ (dark yellow line) and the asymptotic value of $\lim_{\beta_{1}\to \infty }\beta^{\left(s\right)}=1.66098\ldots$ (green line) have been calculated analytically.

**Figure 5:**
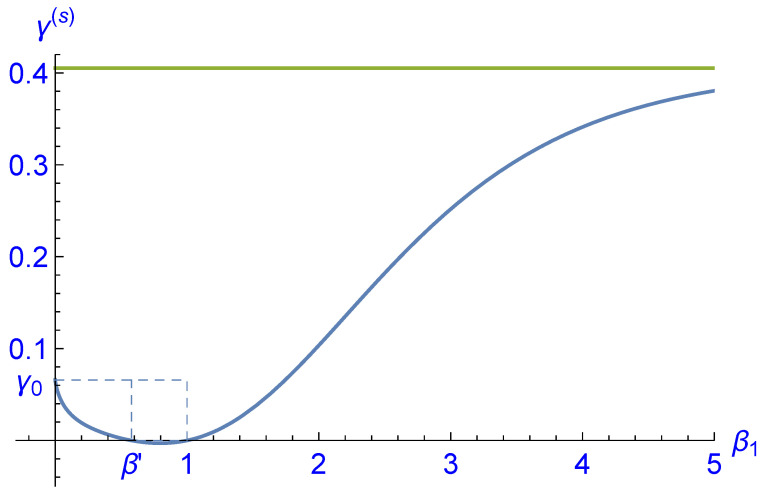
Plot of the second parameter $\gamma^{\left(s\right)}$ characterizing the steady state distribution $p^{\left(s\right)}$ of the analytical example as a function of $\beta_{1}$ for fixed $\beta_{0}=1$ (blue curve). The value of $\gamma^{\left(s\right)}=\gamma_{0}=0.0658087\ldots$ for $\beta_{1}=0$ and the asymptotic value of $\lim_{\beta_{1}\to \infty }\gamma^{\left(s\right)}=0.405262\ldots$ (green line) have been calculated analytically. For $\beta_{1}=\beta_{0}=1$, the parameter $\gamma^{\left(s\right)}$ vanishes because the corresponding Gibbs-type probability distribution is a fixed point of *T*. Interestingly, there exists a second zero of $\gamma^{\left(s\right)}$ at $\beta_{1}=\beta^{\prime }=0.578258\ldots$. In the interval $\beta^{\prime }<\beta_{1}<1$, the parameter $\gamma^{\left(s\right)}$ has small negative values and hence the function $E_{n}\mapsto \log p_{n}^{\left(s\right)}$ is slightly concave.

Next we will investigate the structure of the steady state distribution $p^{\left(s\right)}$ as a function of $\beta_{0}$ and $\beta_{1}$ for the present example. A general probability distribution *p* of a 3-level system with energies $E_{1},E_{0},E_{-1}$ can be characterized by two parameters β,γ such that

(74) $$p_{n}=\frac{1}{Z}\exp\left(-\beta E_{n}+\gamma E_{n}^{2}\right),\;\mathrm{for}n=1,0,-1\;,$$

where *Z* is chosen such that $p_{1}+p_{0}+p_{-1}=1$. A Gibbs-type probability distribution would thus be characterized by γ=0 and β being the inverse temperature of the distribution. It is then a straightforward task to calculate the parameters $\beta^{\left(s\right)},\gamma^{\left(s\right)}$ corresponding to the steady state distribution $p^{\left(s\right)}$ as a function of $\beta_{0}$ and $\beta_{1}$. Again, the result is too complex to be shown, but it can be plotted for fixed $\beta_{0}$, say, $\beta_{0}=1$; see Figure 4 and Figure 5. Surprisingly, we find that for $\beta_{0}=1$ and $\beta_{1}=\beta^{\prime }=0.578258\ldots$, the second parameter $\gamma^{\left(s\right)}$ vanishes and hence the corresponding steady state distribution is of Gibbs type.

## 5. The Linear Regime

So far, we have not made any assumptions about the relationship of the stochastic matrices $T^{\left(0\right)}$ and $T^{\left(1\right)}$. They can be completely independent except that they have Gibbs-type fixed points. If $T^{\left(0\right)}$ and $T^{\left(1\right)}$ were derived from a physical model of the interaction of the NLS with a heat bath of inverse temperature β, as in the example of Section 4.2, there would probably be further relations between $T^{\left(0\right)}$ and $T^{\left(1\right)}$, but we have not needed to consider them so far. However, if we turn to the linear regime where the value of $\delta :=\beta_{1}-\beta_{0}$ is infinitesimally small, we will need the following mild assumption, that is fortunately satisfied for the analytical example of Section 4.2:

**Assumption 1.**  *There exists a smooth family δ↦T(δ) of (left) stochastic matrices defined for all $\left|\delta \right|<r$ and some r>0 such that*

(75) $$T^{\left(0\right)}=T\left(0\right),\;\mathrm{and}\;T^{\left(1\right)}=T\left(\delta \right)=T\left(0\right)+\delta \dot{T}\left(0\right)+O\left(\delta^{2}\right)\;.$$

Here, the overdot $\dot{}$ means ${\frac{\partial }{\partial \delta }\left(\ldots \right)|}_{\delta =0}$. We will henceforward omit the argument δ=0 for sake of simplicity. For example, for the total cycle we have

(76) $$T=T^{\left(1\right)}\;T^{\left(0\right)}=T\left(\delta \right)\;T=T^{2}+\delta \;\dot{T}\;T+O\left(\delta^{2}\right)\;.$$

The steady state distribution $p^{\left(s\right)}$ will also be chosen as a smooth family denoted by p(δ) such that $T\;p^{\left(s\right)}=p^{\left(s\right)}$ now reads

(77) T(δ)Tp(δ)=p(δ),

and

(78) $$p\left(\delta \right)=p+\delta \;\dot{p}+O\left(\delta^{2}\right)\;,$$

where $p=p^{\left(\beta_{0}\right)}$.

We will calculate $\dot{p}$ by means of first order perturbation theory. This is similar in concept to the derivation of a Kubo formula in Liouville space in [7], where the temperature gradient introduced by the two heat baths is treated as an external perturbation. Because the unperturbed matrix will, in general, not be symmetric, some slight modifications of the Rayleigh–Schrödinger perturbation theory known from quantum mechanics are required, which will be derived in what follows. To simplify these calculations, we will restrict ourselves to the “generic case” of

**Assumption 2.**  *The eigenvalue $t_{1}=1$ of T is algebraically non-degenerate and, for the other eigenvalues $t_{2},\ldots$, the algebraic and geometric degeneracy coincides.*

Hence there exists a complete basis of $R^{N}$ of (right) eigenvectors $|e_{n}\rangle$ of T satisfying

(79) $$T\;|e_{n}\rangle =t_{n}\;|e_{n}\rangle \;,$$

for n=1,…,N, where $|e_{1}\rangle =p=p^{\left(\beta_{0}\right)}$ and $t_{1}=1$. Here we have switched to Dirac’s bra–ket style, which makes the notation somewhat easier. The dual basis of left eigenvectors of T will be denoted by $\langle e^{n}|,\;n=1,\ldots ,N$, such that the following holds:

(80) $$\langle e^{n}e_{m}\rangle =\delta_{m}^{n}\;\left(\mathrm{Kronecker}\mathrm{delta}\right)\;,$$

and

(81) $$𝟙=\sum_{n=1}^{N}|e_{n}\rangle \langle e^{n}|\;.$$

The latter implies

(82) $$T=T\;𝟙=\sum_{n=1}^{N}T\;|e_{n}\rangle \langle e^{n}|\overset{\left(79\right)}{=}\sum_{n=1}^{N}t_{n}\;|e_{n}\rangle \langle e^{n}|\;.$$

Obviously, $\langle e^{1}|=\left(1,1,\ldots ,1\right)$. Let ${\langle e^{1}|}^{⊥}$ denote the subspace

(83) $${\langle e^{1}|}^{⊥}:=\left\{x\in R^{N}\left|\langle e^{1}|x\rangle =\sum_{n=1}^{N}x_{n}=0\right.\right\}\;.$$

Upon differentiating (77), we obtain

(84) $$\dot{T}\;T\;p+T^{2}\;\dot{p}=\dot{p}\;,$$

and hence, using Tp=p,

(85) $$\left(𝟙-T^{2}\right)\;\dot{p}=\dot{T}\;p\;.$$

The two vectors $\dot{p}$ and $\dot{T}\;p$ lie in the subspace ${\langle e^{1}|}^{⊥}$, and the latter is left invariant under the linear map $\left(𝟙-T^{2}\right)$. Hence (85) can be solved for $\dot{p}$ by inverting the restriction of $\left(𝟙-T^{2}\right)$ to the subspace ${\langle e^{1}|}^{⊥}$. By means of (82), the result can be written as

(86) $$\dot{p}=\sum_{n=2}^{N}\frac{1}{1-t_{n}^{2}}\;|e_{n}\rangle \langle e^{n}|\dot{T}|p\rangle \;.$$

This completes the calculation of the steady state distribution in linear order in $\delta =\beta_{1}-\beta_{0}$. Next we consider the corresponding energy transfer $\langle \Delta Q\rangle$. Let $E\in R^{N}$ be the “energ” vector with components $\langle n\left|E\right.\rangle =E_{n}$. The expectation value $Q_{0}$ of the energy of the NLS before the contact with the first heat bath is

(87) $$Q_{0}=\sum_{n}p_{n}\left(\delta \right)\;E_{n}=\sum_{n}p_{n}\;E_{n}+\delta \;\sum_{n}{\dot{p}}_{n}\;E_{n}+O\left(\delta^{2}\right)\;.$$

After the contact with the first heat bath and a subsequent energy measurement, we obtain the energy expectation value

(88) $$\begin{array}{ccc} Q_{1} & = & \sum_{n}q_{n}\;E_{n}=\sum_{nm}T_{nm}^{\left(0\right)}\;p_{m}\left(\delta \right)\;E_{n} \end{array}$$

(89) $$\begin{array}{ccc} & = & \sum_{nm}T_{nm}\;p_{m}\;E_{n}+\delta \;\sum_{nm}T_{nm}\;{\dot{p}}_{m}\;E_{n}+O\left(\delta^{2}\right) \end{array}$$

(90) $$\begin{array}{ccc} & = & \sum_{n}p_{n}\;E_{n}+\delta \;\sum_{nm}T_{nm}\;{\dot{p}}_{m}\;E_{n}+O\left(\delta^{2}\right)\;. \end{array}$$

Hence

(91) $$\langle \Delta Q\rangle =Q_{1}-Q_{0}=:\delta \;a+O\left(\delta^{2}\right)$$

where

(92) $$\begin{array}{ccc} a & = & \sum_{nm}T_{nm}\;{\dot{p}}_{m}\;E_{n}-\sum_{n}{\dot{p}}_{n}\;E_{n} \end{array}$$

(93) $$\begin{array}{ccc} & = & \langle E\left|T-𝟙\right|\dot{p}\rangle \end{array}$$

(94) $$\begin{array}{ccc} & \overset{\left(86\right)}{=} & \langle E|\sum_{n=2}^{N}\frac{t_{n}-1}{1-t_{n}^{2}}\;e_{n}\rangle \langle e^{n}|\dot{T}|p\rangle \end{array}$$

(95) $$\begin{array}{ccc} & = & -\sum_{n=2}^{N}\frac{1}{1+t_{n}}\;\langle Ee_{n}\;\rangle \langle e^{n}|\dot{T}|p\rangle \;. \end{array}$$

This completes the calculation of the slope *a* of the tangent of $\beta_{1}\mapsto \langle \Delta Q\rangle$ at $\beta_{1}=\beta_{0}$, which can be considered as a sort of heat conduction coefficient. The fact that a≥0 is a consequence of (55).

This calculation establishes an “external Fourier law”, similarly as in [7], that holds independent of a possibly constant temperature gradient inside the NLS.

In the example of Section 4.2 and for $\beta_{0}=1$, the right and left eigenvectors of T as well as $\dot{T}$ can be explicitly calculated, although they are not shown here due to their overwhelming complexity. Inserting these terms into (95) gives a≈0.173895, the slope of the common tangent of the three function plots in Figure 3. Similarly, in the example of Section 4.1, the slope of the common tangent was calculated as a≈0.249977(N=5), and a≈0.306891(N=100), and plotted in Figure 2.

## 6. Summary and Outlook

The second law of thermodynamics has several formulations of varying generality; one of them is that heat always flows spontaneously from the hotter to the colder body. In the field of quantum mechanics, the formulation, let alone the proof, of a corresponding second law is highly obscure and controversial, despite (or even because of) the large body of literature on this subject. In this situation, it seems advisable to gather a few facts that capture what we know for sure. We know that the von Neumann entropy increases in the course of a Lüders measurement [47]. Further, again in the case of sequential Lüders measurements, there hold various Jarzynski-type equations, and, for each of them, Jensen’s inequality produces second law-like statements; see [30,31]. In this paper, we have singled out a particular Jarzynski-type equation so that the corresponding second law-like statement essentially coincides with the above formulation, namely, that heat flows from the hotter to the colder heat bath. However, the latter was only demonstrated for the collision model we used for heat transfer.

However, although this result is very general and relatively easy to prove, we need to note some restricting assumptions that were required for the proof. The quantum system is an arbitrary *N*-level system (NLS), and we implicitly assume that its states are completely characterized by the occupation probabilities $p_{n}$ of the *n*th energy level, not only at the beginning but also during the whole heat conduction process. The two heat baths are not analyzed in detail; they almost appear as “black boxes” only described by stochastic matrices that contain the conditional transition probabilities between the energy levels of the NLS and leave invariant Gibbs states of inverse temperature $\beta_{i},i=0,1$. For technical reasons, we had to alternate contacts of the NLS with the two heat baths, as we would lose the overview of the heat and entropy transfer with a total contact of all three systems. Hopefully, this special protocol will be a reasonable approximation for real heat conduction processes. As a benefit of our approach, we note that no thermalization assumptions are needed; the heat conduction process can be arbitrarily irreversible. Our calculations are essentially confined to the steady state regime. Of note, the approach to a steady state after a large number of cycles is not an extra assumption but follows from the property that all eigenvalues of a stochastic matrix except the eigenvalue 1 are <1 in absolute value.

It looks promising to apply the present approach to similar problems. For example, a general thermodynamic process could be split into pure heat and work parts that can be represented by (bi-)stochastic matrices analogously as in this paper, and the Jarzynski–Jensen method could be used to obtain second law-like statements.

## Data Availability

No new data were created or analyzed in this study. Data sharing is not applicable to this article.
